# Sustainability and Gender Perspective in Food Innovation: Foods and Food Processing Coproducts as Source of Macro- and Micro-Nutrients for Woman-Fortified Foods

**DOI:** 10.3390/foods11223661

**Published:** 2022-11-16

**Authors:** Estrella Sayas-Barberá, Jose Angel Pérez-Álvarez, Casilda Navarro-Rodríguez de Vera, Manuela Fernández-López, Manuel Viuda-Martos, Juana Fernández-López

**Affiliations:** 1 IPOA Research Group, Agro-Food Technology Department, Centro de Investigación e Innovación Agroalimentaria y Agroambiental (CIAGRO-UMH), Miguel Hernández University, Orihuela, 03312 Alicante, Spain; 2 Servicio de Endocrinología y Nutrición, Hospital Clínico Universitario Virgen de la Arrixaca, Ctra. Madrid-Cartagena s/n, 30120 El Palmar, Spain

**Keywords:** woman, gender-equality, foods, fortification, coproducts, sustainability

## Abstract

Micro- and macro-nutrient deficiencies among women are considered a global issue that the food industry has not adequately considered until recently. The industry must provide and guarantee a diversity of food products worldwide that allow women to get a correct and balanced diet according their life stage. The food industry must focus on this challenge within a framework of sustainable production, minimizing the use of natural resources and avoiding the emission of waste and pollutants throughout the life cycle of food. Food coproducts are presented as potential bioactive functional compounds which can be useful for technological purposes, due to the fact that they can serve as non-chemical, natural and health-improving food ingredients. In this review, we focus on the potential use of food processing coproducts which must be part of a strategy to promote and improve women’s health and well-being. This knowledge will make it possible to select potential ingredients from coproducts to be used in the fortification of foods intended for consumption by females and to introduce sustainability and gender perspectives into food innovation. The attainment of fortifications for foods for women has to be linked to the use of sustainable sources from food coproducts in order to be economically viable and competitive.

## 1. Introduction

Some food components have the ability to prevent or protect against several diseases and affect the specific molecular systems and mechanisms that maintain body functions [1]. Consumers are becoming more and more aware of these aspects and have enhanced their diets and lifestyles, thereby improving their living standards and health.

According to the Sustainable Development Goals (SDG), gender equity and the need for female adults, adolescents and children to receive adequate nutrition are priorities of the 2030 Agenda for Sustainable Development. Yet, women and girls are twice as likely to suffer from malnutrition as men and boys for a combination of biological, social and cultural reasons. Therefore, more effort should be devoted to achieving adequate nutrition for women. One of the best ways to achieving this must be through education and the promotion of programs and interventions that specifically target women at each stage of their lives (adolescents, pregnant women, lactating women and the elderly). This will ensure an adequate quality of life and prevent the occurrence of deficiencies of certain nutrients, thereby preventing problems such as increased risk of injuries, alterations of the menstrual cycle and emotional changes, as well as improving performance in sports.

In recent decades, the importance of a sex-gender perspective in health and nutrition has become notable, especially in the context of cardiovascular diseases and eating disorders [1,2,3,4]. This, along with numerous metabolic sex-gender differences, show the enormous relevance of gender in the field of nutrition [1].

The scientific literature has shown the importance of sex-gender differences in diabetes mellitus (DM), cardiovascular diseases (CVD) and other disease risk factors [5]. Marino et al. [1] showed that nutrition should be viewed through ‘‘gender glasses’’ because of the important metabolic differences that exist between men and women. As such, scientists are required to apply “gender glasses” with regard to health and nutrition [1,6,7].

Women are at a greater risk of presenting nutritional deficiencies, since physiologically, they have greater needs for certain micronutrients such as iron, calcium and folate [6,7]. Deficits of proteins, carbohydrates, iron, vitamin D, calcium, magnesium, group B vitamins, antioxidants, etc. have been found [6,8,9]. Many of these nutritional needs depend on age and other factors, such as, among others, willingness to consume such foods and affordability; associated deficits can lead to reduced well-being, which affects mood, health and performance [8,9,10,11].

These micronutrient deficiencies are more severe in women in resource-poor settings, with a low-quality and monotonous diets [12,13]. In such contexts, it has been shown that micronutrient intake for women of reproductive age is far from adequate [14,15]. Often, the relationships between food group diversity and micronutrient adequacy can differ based on seasonal food availability [14]. In developed countries, dietary diversity is strongly associated with nutrient adequacy [14,15].

Moreover, women’s nutritional requirements change with each life stage, i.e., infancy, puberty-adolescence, sexual maturation (reproductive age), climacteric period and post-climacteric (elderly) years [6,10]. Thus, age or life stage should be considered in association with sex-gender aspects, since the total intake of energy, protein, essential and non-essential amino acids, alcohol, water, sodium and fiber decreases significantly with age, and the absorption of nutrients is subject to fluctuations in the hormonal milieu and circumstances which are specific to woman (pregnancy and childbirth) [10,15,16,17].

Some food-related strategies have been applied to reduce micronutrient deficiencies, i.e., supplementation, fortification and the promotion of dietary changes [8], but these approaches vary in terms of effectiveness, cost, efficiency and sustainability. For example, supplementation is often the preferred option, e.g., with iron. Regarding food fortification, although there are different options on the market, very few are specifically intended to reduce nutritional problems for women. Looking forward, the concept of precision or personalized nutrition is becoming important as a strategy that could contribute to preventing or treating various diseases. In this sense, introducing a sex-gender perspective is one of the most relevant variables and the first step to obtaining precision and personalized nutrition [1,11]. Food bioavailability and metabolism are dependent on physiological sex-gender differences [1].

Moreover, food production is responsible for a large share of greenhouse gas (GHG) emissions [18], so future efforts to approach climate change will require the implementation of strategies to reduce food losses and waste. The sustainable use of agri-food waste, byproducts and coproducts to produce value added products (mainly bioactive compounds such as carotenoids, phenolic compounds, essential oils, vitamins, etc.) will provide economic and environmental benefits, contributing to the sustainability of the food industry and to the circular economy [19]. The aforementioned compounds are considered to be functional ingredients in the food industry due to their health properties, e.g., antioxidant and antimicrobial activities, as well as their abilities to work as natural and sustainable ingredients or to be used as raw material for new products [20,21]. Therefore, food processing coproducts, due to the valuable bioactive functional compounds they contain, can be useful for the production of food fortifications intended for women, especially regarding the current demand for nonchemical, natural, safe and health-improving food components. This is in agreement with the latest recommendations, i.e., that the reduction and prevention of macro- and micro-nutrient deficiencies should be achieved by food-based approaches, focusing on sustainability, sex-gender and sociocultural factors.

Some examples of the viability of food coproducts to satisfy these recommendations are related to the negative iron balance which exists in many women, indicating that women’s diets should include iron-rich foods with high bioavailability [1]. Blood and other byproducts of the meat processing industry are rich in proteins such as fibrinogen, globulins, and albumins, as well as hemoglobin [22,23]. Other components of great relevance are plant-derived flavonoids, which have been recognized for their estrogen-mimetic effects [1]. The enrichment of foods with these compounds can have advantages such as preventing osteoporosis [24,25], reducing the risk of coronary artery disease [26,27] and preventing estrogen deficiency in women during menopause [1]. Plant coproducts are rich in polyphenols that could be used for this purpose [19]. Additionally, bioactive peptides obtained from protein-rich plant byproducts, which present antioxidant, antimicrobial, anticancer, hypocholesterolemic, antihypertensive and immunomodulatory activities, could be used in the development of functional foods [21].

This review focuses on the potential use of foods and food processing coproducts as a strategy to promote and improve women’s health and well-being (SDG 3) through their application in the design of foods specifically intended for them, considering sex-gender-specific aspects (SDG 5) and contributing to the sustainability of the food production system (SDG 12). Sections are included about nutritional requirements according to the life cycle of women, sources of specific nutrients or compounds for the design of foods for populations with specific dietary requirements, major bioactive compounds in vegetal coproducts and their effects on women’s health, and finally, the potential use of food fortifications, obtained as food coproducts, as a strategy to reduce nutrient (micro- and macro-nutrient) deficiencies in woman.

## 2. Nutritional Requirements According to the Life Cycle of Women

### 2.1. The Life Cycle of Women

Human bodies change significantly over time, going through different stages. From a biological point of view, the life stages of a typical woman are divided into infancy, puberty (adolescence), sexual maturation (reproductive age), climacteric period and post-climacteric (elderly) years. It is also worth mentioning unique events, such as pregnancy, delivery and lactation [10].

Infancy, sometimes called childhood, is the period from birth to the age of eight. Puberty is the beginning of adolescence, from age nine to thirteen. Adolescence ranges from age thirteen to eighteen. Other authors defined adolescence as “the period of development that begins at puberty and ends in early adulthood and can be divided into three periods: early adolescence (10–14 years of age), late adolescence (15–19 years of age), and young adulthood (20–24 years of age)” [28].

Reproductive age, i.e., adulthood, is the stage from adolescence to old age. In recent decades, due to increased life expectancy and improved health, the onset of the elderly phase may be delayed until the age of sixty or sixty-five years [29].

### 2.2. Nutrition and the Life Cycle of Women

It is well known that nutrition plays a fundamental role in the health of humans throughout life, but recently, it was discovered that nutrition can affect men and female health in different ways. Until the last decade of the 20th century, all health and nutrition studies, which applied to both men and women, were in fact the result of work done exclusively on men, due to the prevailing social structure and culture. However, it is now known that there are many gender differences that clearly affect the factors associated with nutrition and health [1].

Nutrition is crucial in some stages, such as childhood, adolescence and menopause, and in situations such as pregnancy and breastfeeding [30]. People of all ages need basic nutrients to maintain health, i.e., essential amino acids, carbohydrates, essential fatty acids and several vitamins and minerals. Nutrition and lifestyle before and during pregnancy, lactation, infancy and early childhood have been shown to have long-term effects on the health of the child, including the risk of chronic diseases such as obesity, diabetes and cardiovascular disease [31].

**Figure 1:**
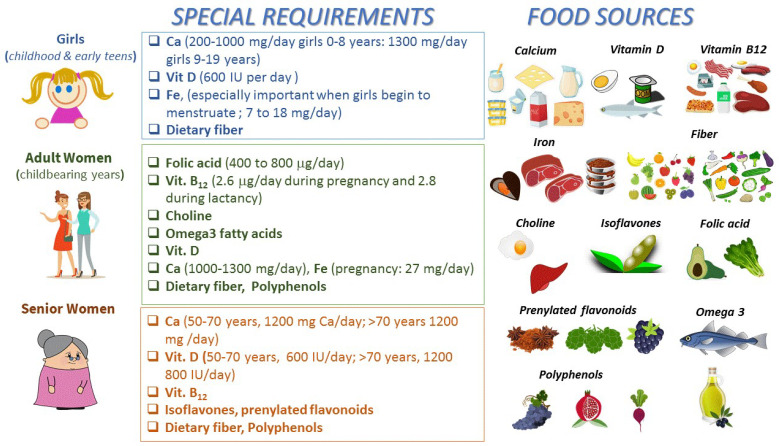
Special nutritional requirements for woman depending on age and physiological state, as well as their main food sources.

Qualitatively, nutritional needs throughout all periods of life are similar, but there are large quantitative differences. For example, during infancy, pregnancy and lactation, the macro- and micro-nutrient requirements are greater than in other phases. For instance, pregnant and lactating women require more energy, protein, iron, calcium and vitamins. Rapid growth during infancy, childhood and adolescence also increases the requirements for essential nutrients. In elderly females, it is important to decrease energy consumption as the basal metabolic rate declines. However, minerals and vitamins, particularly calcium, thiamine and pyridoxine, remain no less essential for the elderly and adults [30]. Special nutritional requirements for women depending on age and physiological state, as well as their main food sources, are summarized in Figure 1.

Apart from macro-minerals (sodium, potassium, chloride, phosphorus, magnesium and calcium), some minerals, including iron, zinc, copper, selenium, iodine, manganese, chromium, molybdenum, boron, vanadium, nickel, silicon and arsenic, are also required in “trace” amounts to maintain physiological functions [32].

These nutrients have an impact on women’s health during their lifetime, but especially during pregnancy and lactation, as well as adolescence and in old age. The intake of micronutrients, such as iron, calcium, iodine and folate, and vitamins A and D, of many women is inadequate. Not only must micronutrient intake be adequate, but also the timing, relative to the life cycle, is important [6].

Good nutrition not only allows proper development to occur at all stages of life; it also prevents diseases such as cancer. Specifically, the control of obesity through nutrition reduces the risk of hormonal breast cancer and, in cases where it has already occurred, of future relapses. In particular, the Mediterranean diet, characterized by an abundance of plant foods and the use of olive oil, plays a favorable role in terms of protecting against cancer; this is attributed to the presence of fiber, vitamins and other bioactive compounds, such as flavonoids, proanthocyanidins and carotenoids [33].

#### 2.2.1. Infancy and Adolescence

Due to the changes and growth that occur during infancy and the health consequences that these have on adults, it is essential to ensure an adequate supply of nutrients to infants. In the first 1000 days of life, growth and nutrition are extremely related. Between 4 months and 2 years, it is critical to identify nutritional deficiencies [34]. Diet not only affects child development in several ways, but also the immune system and neurological and behavioral development [29]. Malnutrition includes both under nutrition and excessive nutrition, that can predispose to obesity, hypertension, diabetes and other diseases later in life. Food intake has to provide enough energy and nutrients to supply for physical activity and permit growth and adequate neurological development. On the other hand, it must minimize the risk of diseases associated with excessive intake, such as diabetes and cardiovascular diseases [34].

The requirements for macro- and micro-nutrients are higher during infancy, proportional to weight, than at any other stage in the human life cycle, except during pregnancy. For almost all infants six months or younger, breast milk is the best source to satisfy nutritional requirements [31]. Recently, milk composition was shown to correlate with the sex of the infant; mothers of male infants produced milk that had 25% more energy than the milk of mothers of female infants. It was also reported that breastfeeding protects girls, but not boys, against viral pneumonia; the susceptibility of non-breastfed females to respiratory viruses has been confirmed [1,29].

After six months, infants can gradually begin to consume solid foods to help meet their nutrition needs [29]. Among the micronutrients that significantly affect children’s development, we can highlight iron, iodine, copper, zinc, folate and choline, calcium, phosphorus and vitamin D. Iron deficiency is associated with poor growth. Iodine is essential for the normal synthesis of thyroid hormone, essential for neurodevelopment. Calcium, phosphorus and vitamin D are essential for adequate bone health and growth. Deficits have been associated with bone weakness, decreased bone mass and poor growth. Reference calcium dietary intakes vary from 200 to 1000 mg/day for children from 0 to 8 years, while those for vitamin D dietary vary from 400 to 600 IU/day for the same ages [34].

There are no known differences in nutritional needs between boys and girls, except for total energy requirements, from the first year of age.

Growth during adolescence requires special nutrient requirements, including amino acids for muscle growth, as well as calcium and vitamin D for bone growth. The energy requirements of adolescent males are higher than those of females. Protein requirements are highest for females in the 11 to 14 year age range, corresponding to the period of greatest growth; additionally, iron requirements are maximal at this time due to menstruation [28].

#### 2.2.2. Adulthood, Pregnancy and Lactation

Women of reproductive age have the same dietary requirements as the general population. Micronutrients are critical for women’s health during the reproductive years. In particular, deficiencies of calcium, iron, folate, zinc, thiamine, riboflavin and vitamins A, D, B-6 and B-12 are very frequent among women of this age [35,36].

Good health and nutrition before conception are crucial to meet the demands of pregnancy and breastfeeding and are vital to the healthy development of the fetus. Many women eat inadequate diets, leading to underweight, obesity and nutrient deficiencies [31].

Human pregnancy has modest nutritional requirements, and most of these can be obtained from a slight increase in a healthy, balanced diet (only about 10–15% extra). Pregnant women should increase caloric intake during the second and third trimesters to cope with the bulk of fetal and placental growth [1,31].

Basic recommendations regarding nutrient supplementation during pregnancy include: iron, to prevent anemia; vitamins A, B_12_ and D, as well as iodine, for women who are at risk of poor supply of these micronutrients; and folic acid to decrease the incidence of neural tube defects in newborns [31,34].

As already stated, malnutrition in women of reproductive age is widespread. This includes undernutrition, as well as excessive consumption of poor-quality, processed foods. During pregnancy, malnutrition is associated with deficiencies of folic acid, vitamin D and iron, resulting in birth defects, impaired fetal development and maternal mortality [36]. Folic acid and iron reduce the risk of congenital defects and pregnancy anemia, respectively. Iodine during pregnancy prevents some childhood cognitive development problems, and vitamin A affects the immune system of the mother and fetus due to the crucial role that it plays in both cellular immune response and humoral immune processes [6].

Adequate energy intake is required to carry out all maternal functions and to ensure adequate tissue deposition and the growth of the fetus. Energy requirements increase in pregnancy to support the major physiological changes that occur, plus the energy required for protein and fat deposition [36].

Additionally, during pregnancy and lactation, the adequacy of certain micronutrients such as iron can have a substantial influence on pregnancy outcome [6]. The recommended dietary allowances for iron during pregnancy are 27 mg/day, i.e., higher than those for adult women or infants, which vary from 18 to 7 mg/day. This makes deficiency of this mineral very frequent during pregnancy. Mild iron deficiency may be asymptomatic, but when depletion becomes severe, anemia, defined as a hemoglobin count of less than 12 g/dL in females, occurs [33]. As with iron, increased zinc requirements during pregnancy (12 mg/day) and lactation (13 mg/day) also increase the risk of zinc deficiency. The same is true for other microelements such as copper (pregnancy: 1 mg/day; lactation: 1.3 mg/day) and iodine (pregnancy: 0.22 mg/day; lactation 0.29 mg/day) [32]. The maternal polyunsaturated fatty acid (PUFA) status during pregnancy is critical for the PUFA status of the newborn [37].

The mother’s nutrition influences the fat concentration, fatty acid composition (including monounsaturated, n-6 and n-3) and immunological properties of breast milk. While a high-carbohydrate diet increases fatty acid concentrations in breastmilk, a high-fat diet results in decreased concentrations. In general, the polyunsaturated fatty acid content in human milk is in accordance with their concentration in the diet of the mother. A deficit of fatty acids in breastmilk can alter the vision and neurological development of the infant [37].

#### 2.2.3. The Elderly

In developed countries, the number of elderly individuals is growing at a rate of about 5% per year. Life expectancy is higher in women than in men, so there is a higher percentage of elderly women than men. Therefore, the nutritional problems of the elderly are mostly women’s problems. This population has higher morbidity than the general population. Nutrition plays an important role in the process of healthy aging. Strategies such as the addition of vitamin D and reducing caloric intake and salt content may extend the life span of elderly women [38].

The risk of undernutrition is prevalent among older patients and is sex-gender related. Female in-patients are at increased risk of undernutrition compared to males [30]. Elderly women eat smaller amounts of food and eat less often than younger women, especially when suffering chronic illnesses, resulting in caloric deficits and malnutrition. The greatest deficiencies occur in proteins, vitamins (B12, D, E) and minerals [38].

Older men and women have basal differences in muscle protein synthesis; the availability of essential amino acids is reduced, and the consumption of extra amino acids cannot overcome this shift [1].

Increased structural instability and fragility of bones occurs at an earlier point in elderly women than in men. This can be explained by the smaller skeletal size and bone mass and the loss of bone mineral density in women, which is accelerated after menopause with the decline in estrogen levels [1].

Sarcopenia is a loss of skeletal muscle mass, quality and quantity, characterized by a slowing of movement, loss of strength and increased risk of injury from falls [39,40]. The prevalence of sarcopenia increased from 13–24% in subjects aged 65 to 70 years to over 50% in those older than 80. With age, prevalence increases in both men and women [3]. Recent advances in nutritional approaches to prevent the development of sarcopenia is mainly focused on the amount and quality of protein intake, the combination of supplements containing amino acids and resistance training, tea catechins, soy isoflavones, and ursolic acid, as well as mild caloric restriction, all of which are effective against age-related muscle atrophy [39,40].

## 3. Macro- and Micro-Nutrients in Foods

Foods are a source of nutrients, energy and other bioactive compounds [41], but not all of them have the same composition. Some foods can be used as sources of specific nutrients or compounds in the design of foods for target populations with specific dietary requirements. It may not always be useful or feasible to consume a certain type of food in its entirety for its macro- and micro-nutrients and bioactive compound content; rather, it may be desirable to extract suitable compounds for the enrichment or fortification of other foods which are more widely consumed or suitable for the age group for which they are intended. 

### 3.1. Plant-Based Foods

In general, plant-based foods are rich in water, carbohydrates and fiber. They have low fat contents, except edible oils, and have no cholesterol. They have proteins in moderate amounts, usually with lower quality than animal proteins, and all types of minerals (although the iron from plant-based foods has lower bioavailability than that in meat) and water-soluble vitamins. Liposoluble vitamins (vitamins E, K and carotenes) can be found in significant amounts in some of these foods. However, retinol and vitamins B12 and D are absent. 

The nutritive value of foods is also affected by other factors, such as how they are consumed (raw or cooked), their processing, interaction with other dietary components, how much and how often they are consumed, the nutritional requirements of each person and the way in which these requirements have been met by other foods in the diet, as well as whether the nutrient in question can be synthetized in the body or not and, etc. [42,43].

In some of these foods (e.g., lentils, potatoes, wheat, corn, rice), starch is the main carbohydrate, while in others (e.g., grape, banana, cherries, sugar cane and sugar beets), mono and disaccharides or simple sugars are most abundant. In peas and corn, the carbohydrates present are initially simple sugars, but these are transformed into starch as they ripe. On the other hand, the starch in unripe fruits such as bananas, apples or pears is converted into sugar as they ripen.

#### 3.1.1. Cereals and Cereal-Based Foods

Cereals are source of complex carbohydrates (70–80%). In general, cereals have a small amount of protein (8–10%; in the case of wheat, gluten is rich in methionine), being lysine and tryptophan their limiting amino acids, which decreases their biological value. Cereals usually have only a small amount of fats [44]. Their dietary fiber content depends on the type of cereal, being higher in whole meal cereals, in which insoluble dietary fiber is the main fraction (cellulose, hemicellulose and lignin). Some cereals such as oats also have a high amount of soluble fiber (mainly B-glucan). Cereals have minerals such as Mg, Zn and Fe and small amounts of Ca; however, the Fe present in cereals has low bioavailability because it is in an inorganic form. In addition, the absorption of Fe can be limited by phytates, which are in the part of the grain where highest levels of minerals are located. Cereals are a good source of B vitamins, i.e., thiamine, B6, folic acid and niacin, but these can be partially eliminated during industrial processing or cooking, especially thiamine and folic acid. As they have no fat, they do not have fat-soluble vitamins, except for wheat germen and corn grains, which contain vitamin E and carotenes. Table 1 shows the composition of the five leading cereal crops in the world (wheat, maize, rice, barley and sorghum), taking into account the fact that the content and composition of nutrients are related to genetic factors and growth conditions.

It is important to note that some of these nutrients can be lost during mincing to obtain flour, which is the main way of using of cereals in food processing (it is the basis for most baked goods, like bread and cookies, as well as pasta) [50]. In this regard, it is important to take into account that the distribution of all these nutrients in the grain is not uniform, with minerals, vitamins and fiber mainly being located in the outer part. So, when the grain is polished to obtain white flour (70–75% extraction), a big part of these nutrients is lost.

It is true that some of these nutrients can be restored (added) after mincing; this is compulsory in white flour in some contexts. Typical nutrients restored through enrichment include iron, folic acid, riboflavin, niacin and thiamin [51]. The main food produced from cereals is bread in its different varieties (white, whole meal, rye, etc.). In general, bread has 30% water and a high carbohydrates content, as starch, (58% in white bread and 49% in whole meal bread) [52]. The energy value of white and whole meal bread is 277 and 258 kcal/100 g, respectively. The protein content of bread is approx. 8% (in wheat bread as gluten), with small amounts of lysine and tryptophan (limiting amino acids, with a consequent decrease in their biological value). However, if cereals are consumed with other foods such as meat, milk, eggs or legumes, the phenomenon of supplementation occurs, causing a notable improvement in the protein quality. Bread can also be made with cereals without gluten such as maize, oat, sorghum or rice, so that it can be consumed by celiac patients [53].

Pasta is also one of the most widespread food products based on with cereal flours; it is a very heterogeneous category, including several types of products which often differ not only in shape but also, and more importantly, in ingredients, and thus, its nutrition composition is highly variable. If the main ingredient used is a type of cereal flour, especially wheat flour, its nutritional value depends on the composition of the wheat. In general, dried pasta (wheat semolina) comprises 70% complex carbohydrates, 3.2% sugars, 13% protein, 3% dietary fiber and 1.5% fat, which means an energy value of 354 kcal/100 g [53,54].

Other foods obtained mainly from cereals are “confectionery products”, which have most of the nutrients reported for cereals but with added fat and sugar; as such, their nutritional value depends on the amount and types added (400–500 kcal/100 g) [52,53].

#### 3.1.2. Vegetables and Fruits

This group includes a wide variety of foods that constitute different parts of plants. For example: spinach, lettuce, endive, chard and parsley are leaves; Brussel sprouts are leafy sprouts; asparagus is tail and leaves; potatoes are tubers; carrots are roots; garlics and onions are bulbs; cauliflower, broccoli and artichoke are flowers; peppers, tomatoes and fruits are fruits; and finally, peas and beans are seeds. However, in spite of this botanical heterogeneity, all of these items show similar nutritional characteristics [52,55,56].

In general, vegetables and fruits have a negligible macronutrient content. The main component is water (7–95%); they have a low protein content (1–5%, and with low biological value) and, in general, they contain virtually no fats (<1%), except for nuts and some fruits (avocado 12%, olives 20% and coconut 36%, which contain mainly monounsaturated fatty acids) or cholesterol [57]. Their dietary fiber (1–5%, both soluble and insoluble) and carbohydrate (5%) contents are also low, except potatoes and bananas, which contain 18% and 20% carbohydrates, respectively, mainly as polysaccharides in potatoes or mono or disaccharides in fruits (fructose, glucose and sucrose). It can be said that tubers and roots have the highest starch content. For this reason, these type of foods are not significant sources of energy (<70 kcal/100 g), with the cited exceptions (potato, banana, avocado and olives). Therefore, based on their low energy contents, big volume and nutrient densities, these foods can be used for slimming diets [56].

The main nutritive value of fruits and vegetables lies in their micronutrient content. They are especially rich in minerals (magnesium, potassium and phosphorous) and water-soluble vitamins (mainly folic acid and vitamins C, A and E) overall when they are freshly consumed (without losses during cooking). Some vegetables (watercress, turnip greens or spinaches) also have high iron contents, but this is not as well absorbed as that from animal-based foods. The same has been reported for calcium which, although it is present in significant quantities in some vegetables (chard, broccoli, watercress, turnip greens or spinaches), some chelators also present in these vegetables, decreasing their bioaccessibility. For example, in the case of spinaches or beets, oxalic acid can bind to calcium, forming calcium complexes that hinder its intestinal absorption [58]. From the fat-soluble vitamins group, the only ones found are vitamin K and carotenes (mainly in fruits and vegetables with strong green, yellow or orange colors). Some fruits also have large amounts of other carotenoid compounds, albeit without provitamin A activity as lycopene (tomato, water melon, cherry, etc.), lutein (chard, celery, broccoli and spinach) and zeaxhantin (spinach and red pepper), which play important roles as protective factors against some degenerative illnesses. In contrast, they do not contain vitamins D, B12 and retinol. Folic acid is found in large amounts in green leafy vegetables (spinach, salads and chard), and vitamin C can be found in all types of fruits and vegetables, especially in peppers, kiwis, strawberries, oranges and mandarins. The quantities of both vitamins can be strongly reduced when the food is cooked or exposed to light [52,55].

In addition to the cited nutrients, vegetables and fruits stand out for their bioactive compounds. Some of the most famous are: capsaicin in hot peppers; flavonols in onions and broccoli; sulfur-compounds in garlic, onions and leeks; glucosinolates in cabbages; carotenoids and chlorophylls in tomatoes, carrots, oranges, papayas, plums, etc.; and organic acids and polyphenols in fruits [59,60].

*Dehydrated fruits and vegetables.* Raisins, date palm fruits, prunes and dried onions, tomatoes, etc. have the same composition as the original fruits but, due to their lower water content, the nutrients are in a higher concentration, increasing their caloric intake. As the drying process may decrease the nutritive value of foods, efforts have been made to develop high-quality dried foods by maximizing their quality attributes such as structure, color, flavor, nutrition, etc. [61,62].

*Nuts* such as almonds, hazelnuts, walnuts and peanuts have low water (10%) and carbohydrate contents (4%), of which starch accounts for approximately 50% and the rest is simple carbohydrates. They have dietary fiber (14%) and protein (20%), although they are a major source of fats (53%), albeit without cholesterol. Therefore, they are a concentrated source of energy (500–600 kcal/100 g). However, fats present are particularly healthy, i.e., mainly monounsaturated and polyunsaturated fatty acids. They also have significant amount of minerals, mainly magnesium (especially in almonds) and potassium, as well as some vitamins such as B6 and E. They do not contain retinol or vitamin B12, D or C [63,64,65,66].

The composition of the five most commonly consumed fruits globally (bananas, melons, apples, oranges and grapes), nuts (peanut, almond, walnut, cashew and pistachio) and vegetables (potatoes, onions, tomatoes, lettuce and peppers) is shown in Table 1.

#### 3.1.3. Legumes

Legumes (common beans, chickpeas, lentils, broad beans, etc.) are very complete foods because they contain almost all the nutrients we need; however, their consumption has drastically decreased (21 g/person/day), maybe due to their loss of prestige in developed countries [67]. Legumes are an important source of protein, dietary fiber, complex carbohydrates, iron, zinc, B complex vitamins and essential amino acids and are practically free of saturated fats [68,69]. They have a low water content (9%), which contributes to their easy preservation. They are an excellent source of protein (24%), with high quality, i.e., very similar to animal protein. Actually, they only lack the amino acid methionine (mainly found in cereals and animal food products), but they are rich in lysine, which is a limiting amino acid in cereals. Carbohydrates are also abundant (>50%), mainly as starch (>90%). Additionally, they contain stachyose and raffinose (glucose + fructose + galactose), which are not digested by intestinal enzymes, and so they arrive to the colon where they are fermented by microbiota, producing acids and gases, causing flatulence. They have significant amounts of dietary fiber, mainly soluble (25% in beans or 12% in lentils). In some cases, these amounts amount can be reduced by soaking and cooking [70]. It is also important to note their low fat contents (2–5%), with polyunsaturated and monounsaturated fatty acids being the predominant ones. Their energy content is not overly high: 300 kcal/100 g as a raw food. Legumes can be considered a good source of minerals (Ca, Mg, Zn, K, P and Fe), and they contain almost all vitamins (B1, niacin, folic acid, carotenes, small amount of B2 and C) but, like all vegetables, they do not contain vitamin B12, retinol or D. Table 1 shows the composition of the five leading legumes in the world.

#### 3.1.4. Vegetable Oils and Fats

In this group, several edible oils and fats have been included which are directly obtained from vegetables or after a technological process. They are used in the production of fried and processed foods, as well as salad oils.

*Olive oil* is the hallmark of the Mediterranean diet, contributing between one-third and two-thirds of the total vegetable fat consumed [71]. It has a high MUFA content, mainly oleic acid (80–90%), but in this case, other minor compounds (with antioxidant functions) such us polyphenols, tocopherols, tocotrienols or beta-carotene are responsible for some of the healthy properties attributed to its consumption [72,73].

*Vegetables oils from oilseeds* are also source of fats (without cholesterol) and vitamin E. Their fatty acid compositions depend on the type of seed from which they are extracted. The major world vegetable oils (palm, soybean, canola, sunflower and peanut oils) contain primarily five C16 and C18 saturated, monounsaturated, and polyunsaturated fatty acids: palmitic acid (16:0), stearic acid (18:0), oleic acid (18:1Δ9), linoleic acid (18:2Δ9,12) and α-linolenic acid (18:3Δ9,12,15) [74].There are other vegetable oils (tropical oils) with a high SFA content (coconut, palm or palm kernel) [75]. Vegetable oils, however, contain a range of phytochemicals, e.g., they are the main source of natural plant sterols in the diet. Plant sterols can be present as free or esterified forms, and the proportions vary, e.g., free sterols dominate in soybean and sunflower oil, while in canola and corn oil, free sterols account for only 30% of the total. Refining vegetable oils (the main way in which they are consumed) decreases the sterol content (by between 10 and 70%, depending on the oil and processing conditions) [76].

*Margarines* produced by the hydrogenation of vegetable oils to make them solid at room temperature, which is a very appreciated attribute (technologically and sensorially), are used in processed foods, as substitutes for SFA (butter). The main problem with this is the formation of trans fatty acids during the hydrogenation process, the consumption of which is a risk factor for cardiovascular diseases. These foods are concentrated sources of energy (899 kcal/100 g), because fats are the predominant nutrient (84%). Some of them contain essential fatty acids (linoleic and linolenic fatty acids), and they are also vehicles for fat-soluble vitamins (mainly vitamins A and E) [77].

### 3.2. Animal-Based Foods

#### 3.2.1. Milk and Dairy Products

Milk is considered one of the most complete foods because it contains almost all the essential nutrients for humans. However, it does not contain vitamin C, dietary fiber or iron. Milk is a nutrient-dense food with important nutritional value due to its calcium, vitamin D, protein, vitamin B12, vitamin A, riboflavin, potassium and phosphorous contents (Table 2). In contrast to other foods of animal origin, milk has a significant amount of carbohydrates (5%), as lactose, which favors calcium absorption. The lack of lactase (the enzyme responsible for its hydrolysis during digestion) in some population groups is responsible for lactose intolerant disorder [78]. Milk has high quality proteins (3.3%) that possess a wide range of nutritional, functional and physiological activities [79]. It is also a unique source of peptides with biological activity, derived from caseins (80%, the main protein fraction) and whey proteins (20%). Bovine milk protein is considered a high-quality, or complete protein, because it contains all nine of the essential amino acids in proportions resembling our amino acid requirements [80]. Its low-fat content (3.7% in whole milk) mainly comprises saturated fat. Milk and dairy products are a good source of vitamins, i.e., mainly retinol and riboflavin. They contain almost all minerals (except iron), especially calcium and phosphorus. Dairy products are the foods with the highest calcium content, which means that if, in some cases, they cannot be consumed (due to allergies, intolerance, etc.) it is difficult to meet the needs for calcium intake. In addition, dairy calcium is better absorbed due to the presence of lactose, vitamin D and the correct proportion of calcium/phosphorus in milk [81]. The main differences between whole milk (3.7% fat), semi-skimmed milk (1.5–1.8% fat) and skimmed milk (<1% fat) are the fat, cholesterol and fat-soluble vitamin contents (Table 2). Skimmed milk (<1% fat) provides less energy but higher nutrient concentrations, and so its consumption can be beneficial for people that, for various reasons (cardiovascular diseases, slimming diets, etc.), want to decrease their fat consumption.

*Yogurt* is one of the most well-known and widely consumed dairy products. It is a fermented food obtained by the action of selected lactic bacteria (*Streptococcus termophilus* and *Lactobacillus bulgaricus*), which act on lactose, producing lactic acid [85]. Therefore, yogurt has less lactose than the original milk and can be a good option for people who are lactose intolerant. A partial lactose hydrolysis (30–70%) can be enough to solve this problem that affects more and more people every day. Its nutritive value is similar to that of the original milk, except for the fact that sugars, fats and other components (fruits, nuts and so on) are often added, increasing its energy value [86].

*Cheese* is produced by milk coagulation with serum elimination; as a result, most of vitamins from the B group, as well as the lactose, are lost. All other milk compounds (proteins, fat, vitamin A and most of the calcium) are present in curd cheese. There are a lot of cheese types, depending on the type of milk used, the water content, the microorganisms used for maturation, fat content, etc. Their compositions depend on the maturation grade, because it is during this process that the amount of water decreases, and therefore, the proportion of other nutrients (proteins and fats) increases. In the end, it can be said that cured cheeses contain water, protein and fat in the same proportion. Their cholesterol content is very variable (80–100 mg/100 g) [87]. Of course, there are also cheeses produced from skimmed milk, with the corresponding composition.

Some of the advantages attributed to the consumption of dairy products are their protein, calcium, retinol, vitamin B2, B1 and B12, folic acid, niacin, magnesium, Zn and phosphorous contents; their easy use and preservation; most of them are easy to chew (which may be very important in some population groups); they can contain up to 80% water; and finally, skimmed milk, except for its lack of liposoluble vitamins, is one of the foods with the highest energy density [88].

#### 3.2.2. Meat and Meat Products

Meat composition depend on the animal from which it is derived and the part of the carcass (muscle). In general, meat has a high water content (>60%) and does not contain dietary fiber or carbohydrates. Meat is a good source of high-quality proteins (20%; approx. 40% essential amino acids), although this depends on the age of the animal; meat from older animals contains more connective tissue and less methionine and other essential amino acids [89]. While meat is a pivotal source of essential amino acids, it also supplies amino acids, amino-acid-derived metabolites and peptides, which have important bioactive properties. Thus, it has been proposed that taurine, creatine, hydroxyproline, carnosine and anserine, which are all mainly obtained from meat, exert important physiological functions [90]. Its energy content (250 Kcal/100 g) mainly depends on the amount of fat, which is highly variable (quantity and quality), which, in turn, depends on the age, sex, feed and carcass part of the slaughtered animal. Lean meats contain between one-third and one quarter the fat (<10%) of fatty meats. In addition, the fat content can be higher in some meat products, such as sausages (>60%). Pork back fat has 71% fat and bacon 47%. In contrast, poultry meat has the lowest fat content. Notably, some of this fat is visible and so can be easily removed before consumption. About fat quality, in general, half are SFA (mainly palmitic and stearic acid) and the other half are UFA, with MUFA being the predominant ones (mainly oleic acid in pork meat) [80,91]. The cholesterol contents of meats range from 30 to 90 mg/100 g. Bone marrow and organs, such as liver, kidney or brain, contain a much higher cholesterol content, up to several hundred milligrams per 100 g. Processed meat products contain from less than 50 mg/100 g to more than 150 mg/100 g, depending on their formulation [92]. Meat is a good source of minerals and vitamins, especially the viscera where they are stored. Meat is rich in iron and zinc that have high bioavailability. Blood, liver and meat products with these things added are the richest sources of iron. Iron in meat is complexed in the form of heme-iron, which has high bioavailability in comparison with inorganic iron (non-heme-iron) from vegetables. It is also important to highlight that meat amino acids increase non-heme iron (from cereals or legumes) absorption [90]. Regarding zinc, its bioavailability is also increased by the presence of meat proteins. Significant amounts of other minerals, such as cooper, magnesium, selenium, phosphorous and chrome, are also found in meats. Meats also have significant amount of vitamins, mainly from the B group (except for folic acid, which is found in significant amounts only in liver), such as B1, B3, B6 and B12 and retinol, with the latter two only occurring in animal-based foods [89]. Meats also have small amounts of other vitamins such as vitamin E, biotin and pantothenic acid. Pork is especially rich in thiamine (B1). Meat has no vitamin C or carotenes [93].

Regarding meat products, their nutritional value depends on the type and amount of meat (lean or fat) used and, overall, on the additives and technologies used for their preservation (i.e., drying, curing, fermentation, heat treatment or smoking). Nearly all processed meat products are cured, which means that salt is added and, in most cases, nitrite or nitrates. First of all, as a consequence of dehydration, nutrient density notably increases in meat products. The extensive proteolysis that occurs during curing-maturation, as a result of endogenous and microbial proteases, leads to high levels of free amino acids (easily digested) and peptides with bioactive activities. Naturally occurring bacteria in sausages, or even used as starters, are mostly strains of lactic acid bacteria with a high degree of hydrophobicity, which usually is linked to probiotic potential. On the negative side, the high salt content and the presence of nitrites in this type of meat products have identified as potential causative factors for some illnesses [90].

#### 3.2.3. Fish and Seafoods

Fish is a valuable source of essential nutrients, especially high-quality protein and fats (macronutrients), as well as minerals and vitamins (micronutrients). Fish contains 18–20% protein (easily digested and with high biological value) and eight essential amino acids, including sulfur containing lysine, methionine and cysteine [94]. It also has a non-protein nitrogen fraction (9–38%) that plays a major role in its quality (deterioration and flavor). The major components of this fraction are volatile bases, guanidine compounds, free amino acids, nucleotides, urea and betaines. Depending on their nutritional profile (mainly related to fat content), fish can be classified in fatty, lean and semi-fatty. Fatty fish store the fat mainly in the muscle, while lean fish do so in liver, with only a very small present in muscle. Fatty or blue fish has 8–16% fat content (i.e., sardine, bonito, herring, mackerel and salmon). Lean or white fish has <1–3% fat content (i.e., cod, whiting, pike, monkfish, sole). Semi-fatty fish has 3–6% fat content (i.e., trout, red mullet, and turbot). In general, it can be said that even fatty fish has a lower fat content than meat, and so its energy values are lower (lean fish: 60–80 kcal/100 g; fatty fish: 150–200 kcal/100 g). Fish fat is one of the most unsaturated fats in the animal kingdom. Fish is an important source of dietary n-3 PUFA (long-chain fatty acids). The most typical fatty acids found in fish are eicosapentaenoic (EPA) and docosahexaenoic (DHA) fatty acids, both of which have important antithrombotic and anti-inflammatory activity; as such, the consumption of fish is seen as beneficial in terms of reducing the risk of cardiovascular diseases. However, both the amount and the composition of fat in fish can change depending on several factors, i.e., the use of spices, the age, sexual state and seasonality of the fish, the plactonic richness in the sea, temperature, farming conditions and the processing and cooking technique [95]. In this regard, it has been reported that farmed fish have a different FA profile to wild fish due to the incorporation of vegetable oils and other vegetable materials containing fat in their diets. Indeed, their FA profile shows high levels of oleic acid as well as n-6 PUFA and is less rich in EPA and DHA [96]. Fish is rich in calcium (mainly if bones are eaten, as with young or canned fish), potassium, zinc, phosphorus, fluor, selenium, iodine and iron, and is a vital source of vitamins, especially vitamins A and D, as well as thiamine, riboflavin, and niacin (vitamins B1, B2, and B3, respectively). The vitamin A found in fish is more bioavailable compared to that in plant foods; also, fatty fish contains more vitamin A than the lean types. Vitamin D can be found in fish liver and oils [94,97]. Some fish (salmonid), lobsters and marine crustaceans also contain carotenoid compounds (astaxanthin and fucoxanthin), which have interesting biological properties.

*Shellfish and crustaceans* have more or less the same composition as fish but with lower fat and higher cholesterol contents. For example, crustaceans contain cholesterol at relatively high levels compared to muscle meats (e.g., >120 mg/100 g in steamed lobster vs. 82 mg/100 g in 90% cooked lean ground beef) [98]. As for minerals, zinc and iron levels are similar to those of meat.

A negative aspect of fish consumption is its vulnerability to contamination by pollutants such as heavy metals (mainly Hg, but also Pb, Cd, Cr and As). Fish accumulate heavy metals by uptake through the gills and the skin and can bioaccumulate and bio-magnify them to toxic levels for human consumption [99].

#### 3.2.4. Eggs

Eggs are an important source of almost all nutrients, dietary fiber, vitamin C and carotenes. They contain vitamin D, E B12, retinol and riboflavin, as well as iodine and iron. It must be noted that while some of these components are distributed equally between the egg white and yolk, lipids, vitamins and minerals are mainly concentrated in the yolk. In general, egg white is a mix of water (88%) and protein (11%). In contrast, egg yolk has a lower water content (50%) but higher fat (33%) and protein (16%) contents, and it is an important source of vitamin D and lutein (a bioactive compound which is important for eye health). It is true that while some egg components, mainly minerals, vitamins and fatty acids, are highly influenced by hen nutrition, others (major constituents) are very stable [100,101].

Egg proteins (13%) are highly digestible, although a small amount of them are not assimilated, mainly when egg is consumed raw. Nearly 1000 different proteins have been identified in chicken eggs. Ovalbumin is the main protein in egg white, representing a valuable source of amino acids for human nutrition. In addition, four highly abundant protease inhibitors found in egg white may delay the digestion of egg components, especially when eggs are consumed raw, because they are easily inactivated by heat treatment. Egg yolk has a high proportion of lipoproteins (LDL and HDL) immunoglobulins and other soluble proteins [102].

The fat content (12%) is concentrated exclusively in the yolk, as part of the yolk lipoproteins. Its composition is 5.3 g/100 g of UFA (MUFA + PUFA) and 2.6 g/100 g of SFA. Yolk is also a rich source of essential fatty acids such as linoleic acid. The cholesterol content is mainly found in the yolk (400 mg/100 g of whole egg). Eggs contain vitamin D, E B12, retinol, riboflavin, iodine and iron [103].

The carbohydrate content in eggs is very low (0.7%), distributed between the egg white and yolk. The dominant free sugar in eggs is glucose, which is mainly found in egg white.

Egg yolk can be considered a vitamin-rich food because it contains all vitamins except vitamin C, with the most abundant vitamins being A, D, E, K, B1, B2, B5, B6, B9 and B12. However, the amount of fat-soluble vitamins (A, D, E, K) in egg yolk is highly dependent on hen feeding. In contrast, the most abundant vitamins in egg white are B2, B3 and B5. In addition to their vitamin content, eggs can also be considered a relevant source of choline, which is especially concentrated in the yolk (680 mg/100 g egg yolk). Choline plays important roles in neurotransmission, brain development and bone integrity, among others [104]. It is important to note that in spite of this high amount of vitamins, eggs contains some vitamin-binging proteins, which can limit access to those vitamins. One such examples is avidin, which can bind to vitamin B12, decreasing its bioavailability. The most abundant minerals in eggs are P, Ca and K. In addition, the amount of iron and zinc found in egg yolk is very significant [101]. Due to the easy modification of the amount of some nutritive compounds in eggs by changes in hen’s diet, it is common to find enriched eggs in the market, mainly in terms of the n-3 fatty acid, vitamin E, carotenoid and selenium contents [105].

## 4. Major Bioactive Compounds Found in Vegetal Coproducts and Their Effect on Women’s Health

Every year, a huge volume of biowaste, including byproducts, coproducts and biomass residues, is created from the agri-food industries. These coproducts are often a great source of bioactive compounds, i.e., mainly dietary fiber, proteins and polysaccharides, as well as phytochemicals, which are generally intended for use as animal feed or are used for composting. Within a circular economy model, these coproducts can be exploited in different sectors to obtain compounds with a high-added value. One such sector would be the food industry, that could use them as potential ingredients in the development of functional foods. A sector of the population to which foods fortified with phytochemical compounds extracted from agri-food industry coproducts could be directed, is women, especially in the pregnancy, premenstrual and menopause periods. These periods are profoundly affected by hormone levels, mainly estrogen concentrations.

### 4.1. Isoflavones

Several studies have reported that some phytochemicals, like isoflavones, showed in vitro estrogenic activity [106,107,108]. Thus, the positive effects of isoflavones on osteoporosis, as well as on the alleviation of postmenopausal syndrome, have been widely recognized [109,110]. In general terms, the administration of dietary supplements or foods rich in isoflavones yielded a moderate reduction in the incidence of hot flashes (10–20%) [111]. In the same way, Ferreira et al. [112] reported that a higher dietary intake of isoflavones was associated with a lower risk of subclinical cardiovascular disease in postmenopausal women. Finally, several epidemiological studies have suggested that the consumption of isoflavones may be associated with lower incidence of osteoporotic disorders [113,114].

Isoflavones only occur in a limited number of plants, primarily in soybeans (Glycine max), and therefore, in soy-derived food. Additionally, high contents of isoflavones are found in red clover (Trifolium pratense), white clover (Trifolium repens) and alfalfa (Medicago sativa) [115]. Soybean cake is a coproduct generated in the soy oil industry. It is rich in protein, polysaccharides and phytochemicals including polyphenolic compounds; despite this, it is underutilized as a food ingredient [116]. As mentioned above, soybean coproducts are a great source of bioactive compounds including isoflavones. Thus, Kao et al. [117] analyzed the isoflavone content of soybean cake obtained from the oil industry. It was reported that this coproduct was rich in genistin, glycitin and daidzin, with values of 1224, 356 and 421 mg/kg, respectively. Tyug et al. [118] carried out a study to determine the isoflavone content of soy coproducts. It was found that in soymilk powder, the main isoflavones were daidzein (31.3 mg/kg wet weight) and genistein (8.40 mg/kg wet weight), whilst in soy husk powder, only daidzein was identified; it was quantified with a value of 11.7 daidzein/kg wet weight. More recently, Jankowiak et al. [119] investigated the extraction of isoflavones from crude okara, a coproduct from soymilk production. Those authors found that the total isoflavone content ranged between 849.9 and 923.4 mg/kg. Tangkhawanit and Siriamornpun [120] reported that the main isoflavones found in soybean cake from the oil industry were daidzein and genistein, with values ranging between 63.41 and 463.14 mg/kg and 21.05 and 84.07 mg/kg, respectively.

### 4.2. Prenylated Flavonoids

Several compounds, including 8-prenylnaringenin (8-PN), 6-prenylnaringenin (6-PN) and xanthohumol, have demonstrated strong estrogenic activities [121]. The high estrogenic activity of 8-prenylnaringenin and 6-prenylnaringenin is due to the ability of these molecules to interact with estrogen receptors [122]. The effects of prenylated flavonoids, mainly 8-PN and 6-PN, and their effectiveness in reducing menopause-associated problems, including hot flashes, osteoporosis and cardiovascular disease, have been analyzed in several works [123,124].

A major source of 8-prenylnaringenin, 6-prenylnaringenin and xanthohumol is hop cones (*Humulus lupulus* L.), which are used to obtain the desired organoleptic properties of beers, including its bitterness and aroma, as well as to improve its conservation [125]. Several studies have noted that a great portion of hops are being wasted and treated as coproduct materials in the brewing industry [126]. These coproducts are rich in bioactive compounds and have potential uses. For example, 8-prenylnaringenin has been identified at a concentration of 0.002% of the dry matter of hops [127], whilst 6-prenylnaringenin was found in slightly higher amounts (0.01%) [128].

### 4.3. Dietary Fibre

Constipation is a common disorder in post-menopausal women. It is possibly the result of a reduction of hormone levels including estrogen and progesterone [129]. Similarly, constipation during pregnancy is attributed to the rise in progesterone hormones which relax the intestinal muscle [130]. Some commonly recommended dietary changes for the treatment of constipation are to increase the ingestion of fluids and fiber [131]. The coproducts generated in the industrialization of fruits and vegetables are very important sources of dietary fiber, which can be categorized into insoluble dietary fiber (IDF) and soluble dietary fiber (SDF). These coproducts could be used as potential ingredients in the development of functional foods which are rich in dietary fiber. In this sense, Lucas-Gonzalez et al. [132] reported that the total dietary fiber content in coproducts from kaki (Diospyros kaki Thumb) of cv. “Rojo brillante” was 37.07 g/100 g dry weight (dw), with an IDF content of 25.30 g/100 g. Silva et al. [133] analyzed the dietary fiber content of tomato pomace, a coproduct generated during tomato processing, mainly comprising tomato skin. Those authors found that among the nutrients present, the most abundant was dietary fiber, with values ranging between 48.62 and 53.97 g/100 g dw. Wang et al. [134] studied the total dietary fiber content of apple pomace (consisting of peel, pulp and seeds), a coproduct obtained from the cider-making process. It was reported that this coproduct showed a total dietary fiber content of 26.6 g/100 g dw, with 18.4 g/100 g of IDF and 8.2/100 g of SDF. More recently, Botella-Martinez et al. [135] carried out a study to analyze the dietary fiber content of flour obtained from cocoa bean (*Theobroma cacao* L.) shells, a coproduct of the chocolate industry. Values of between 61.18 and 65.58 g/100 g dw were found, depending of the particle size. In a similar study, Belmiro et al. [136] analyzed the dietary fiber content of coffee coproducts, i.e., the husk, pulp, parchment and silverskin. It reported that coffee coproducts are a rich source of dietary fiber, with a content of 63.0 g/100 g dw. This fiber comprised 58 g/100 g dw of IDF and 5 g/100 g dw of SDF.

### 4.4. Polyphenols

During the nine months of a typical pregnancy, three immunological phases can be clearly differentiated, which coincide with the first, second and third trimesters. The first and third trimesters are pro-inflammatory phases due to the insults caused by blastocyst implantation and parturition, respectively. On the other hand, the second trimester represents a predominantly anti-inflammatory state [137]. In this sense, a high intake of foods with high contents of polyphenol compounds (phenolic acids, flavonoids, stilbenes and lignans) has been related with a reduction in the incidence of chronic inflammatory diseases [138,139,140]. The coproducts generated in the agri-food industry, and mainly in the fruit processing industry, are a great source of polyphenolic compounds. Therefore, Pintać et al. [141] carried out a study to analyze the total phenol and flavonoid contents of grape pomace, which is a winemaking coproduct. The former ranged between 34.4 and 77.8 mg of gallic acid equivalents (GAE)/g sample (dw), depending of the grape variety, while the latter varied from 17 mg to 17.4 mg of quercetin equivalent (QE)/g sample (dw). In a similar work, Cheaib et al. [142] analyzed the total phenolic and flavonoid contents of pomace (pressed skins and pulp residues) generated from the industrialization of apricots to obtain purees and jams. A total phenol content of 10.8 mg GAE/g sample (dw) and total flavonoid content of 6.3 mg catechin equivalent/g sample (dw) were reported. Lorente-Mento et al. [143] analyzed the polyphenolic content (total phenolic and total flavonoids) of almond skin vars. “comuna” (ASFC) and “fritz” (ASFF) coproducts produced in the Turrón industry. It was reported that ASFC had a total phenolic content of 6.39 mg GAE/g sample (dw), while ASFF showed a total phenolic content of 1.64 mg GAE/g sample (dw). With regard to the total flavonoids, values of 17.08 and 8.23 mg RE/g sample (dw) were reported for ASFC and ASFF, respectively. More recently, Delgado-Ospina et al. [144] analyzed de total phenolic and flavonoid contents of cocoa shell and cocoa pod husk, two of the most important coproducts of the cocoa agro-industrial chain. They found that in cocoa shell, the total phenolic and flavonoid contents were 9.53 mg GAE/g sample (dw) and 3.21 mg rutin equivalent (RE)/g sample (dw), respectively, while in cocoa pod husk, these values were 16.6 mg GAE/g sample (dw) and 8.4 mg RE/g sample (dw), respectively.

### 4.5. Folic Acid

The preconceptional period has a critical influence on adverse pregnancy outcomes. Insufficient folic acid ingestion during prepregnancy, as well as during the pregnancy, is linked with intrauterine growth retardation, preterm abortions and neural tube defects [145]. At present, the fortification of several foods with folic acid seems to be an initial attempt to design a strategy for the use the functional foods for the prevention or treatment of several disorders caused by folic acid deficit [146]. As with other phytochemicals, the coproducts generated by agri-food industries could be a great source of folic acid to fortify several foods. Leafy vegetables, e.g., spinach, and their coproducts are the main sources folic acid. Jiraungkoorskul [147] reported that the folic content of spinach was 194 µg/100 g, while Saubade et al. [148] reported a value of 200 µg/100 g. Another major source of folic acid is lettuce; Islam et al. [149] reported that stem lettuce is a major source of the nutrient. Those authors found a total folate value of 57.7 µg/100 g FW, while Kim et al. [150] noted that the total folate contents in various lettuce cultivars were in the range of 66.1 (cv. Caesar Green) to 97.3 μg/100 g (dw) (cv. Asia Heuk Romaine).

## 5. Food Fortification as a Strategy to Reduce Nutrient Deficiencies in Women

**Figure 2:**
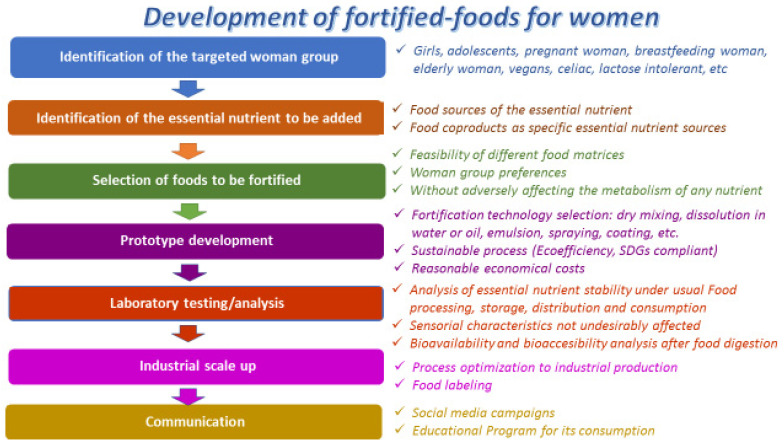
Main steps in the development of fortified foods for women.

Food fortification is the act of adding an ingredient (bioactive compounds, macro- and micro-nutrients) to conventional foods with the aim of enriching and improving its availability or concentration in that food. With fortification, a food can become a functional food. In the case of women, and to meet the nutritional requirements specific to pregnancy or lactation, fortification can be essential. Fortifying conventional foods is an accepted strategy by scientists, food manufacturers and consumers [151,152]. As previously discussed, the sources of bioactive compounds for the fortification of foods can be food coproducts. However, food fortification is not only the addition of bioactive compounds (dietary fibers, vitamins, and minerals, among others); it must be associated with Food Product Development and Innovation (FPDI) strategies. The main steps in the development of fortified foods for women are shown in Figure 2.

Traditionally, FPDI was based only on scientific and technological issues; however, this has changed, and currently, other non-traditional aspects are being included [153]. In the 21st century, it is necessary to have a comprehensive, integral vision of food for an appropriate FPDI strategy [154]. The designed foods would not only have to cover the specific physiological needs of women at different ages, but would also have to take into account anthropological, social, identity, cultural, gastronomic, religious, gender equity, communication, aesthetics and scientific/technological points of view [155,156]. Thus, fortified foods intended for consumption by women are now included in the Research, Development, Innovation, and Communication (R & D & I & C) area. In multicultural societies, the lifestyles of women are directly related to FPDI fortified foods [157]. Although all these points of view are very important for the FPDI of such foods, it must not be forgotten that one of the main aspects of any industry (including the food industry) is to be economically profitable. Competitivity in the business of producing fortified foods for women is essential. In this sense, the use of compounds from food coproducts as sources of fortifications could be more economically viable than the use of other sources.

Such fortified foods must have physicochemical, techno-functional and sensory properties which are close to those of the original food products. This means that these products should mimic the appearance, texture, mouthfeel and flavor, and exceed the nutritional profiles, of the original ones [158] without decreasing their shelf-life. It should also not be forgotten that nutrition programs have the potential to improve women’s health, which is associated with the empowerment of women [159].

Kitesaa et al. [160] reported that in order to successfully design fortified foods for women, it is absolutely necessary to understand the population group to which such foods are addressed (women, children, millennials, vegans, vegetarians, etc.) regarding nutritional requirements, the most common deficiencies and food preferences.

Many fortified foods for women make use of plant-based proteins, polyphenols, vitamin D, calcium and iron, among others. However, the addition of some of these compounds to different food matrices may not be technologically feasible [160,161] for the following reasons: (i) the necessary concentrations to have a physiological effect are very high and the food matrices are affected by such additions (for example, in the case of different types of polyphenols, which are incompatible with many protein systems, like emulsions); (ii) their addition makes the elaboration process unfeasible, affecting techno-functional properties of the fortified food (water and oil holding capacity, swelling capacity) [162]; (iii) fortifications can reduce stability during shelf-life; (iv) aggregations, precipitations and color changes can occur. For example, some sensorial problems have been detected with the addition of certain minerals such as calcium, which causes a “sandy” mouthfeel, or other texture problems in fortified ice creams [163] or unacceptable changes in color or flavor by the addition of iron [164]; and (v) effects on the bioavailability, bioaccessibility and stability of bioactive compounds during digestion, depending on the food matrix. Hurrell [164] reported problems with iron absorption due to the presence of potent inhibitors in some iron-fortified foods. Lynch et al. [165] reported experimental evidence of the considerable variation in the bioavailability of different commercial bioactive compounds such as iron-rich powders, while other authors [166,167,168,169] have reported variations in the bioavailability of polyphenols, depending on the food matrix. To improve the shelf-life, bioavailability and bioefficacy [170] of these types of compounds in fortified foods, different delivery systems have been applied, such as emulsion-based systems (liposome, nanoemulsion, double emulsion and Pickering emulsion) [171] and nano/microparticles-based systems (protein-based, carbohydrate-based and bi-polymer based). Kaushalya and Gunathilake [172] noted that the encapsulation approach for the delivery of bioactive compounds such as polyphenols and micronutrients [173] may be feasible and effective.

An interesting new strategy that can be applied in fortified foods intended for women is the control of the rate of lipid digestion and absorption [174,175]. Modulating the kinetics of lipid digestion and absorption offers interesting possibilities for developing fortified foods that can make it possible to control postprandial lipemia and the release of lipophilic compounds, such as omega-3 fatty acids, liposoluble vitamins, phytosterols and carotenes, among others [176]. Most foods are emulsions, and therefore, can be designed to achieve considerable differences in the kinetics of lipid digestion. Thus, it would be possible to delay/control the digestion of lipids if the stomach emptying rate of such compounds can be altered; this is largely affected by interactions of emulsion droplets with food matrices [177]. However, this strategy has technological problems related to the use of plant-based proteins, polyphenols, phytosterols and liposoluble vitamins [178], as colloidal complexes may form, stabilizing oil-in-water emulsions. Although these emulsions are feasible from a technological standpoint, several problems have been identified (small mean droplet diameters (~200 nm), strong negative surface potentials (~−60 mV), decreased isoelectric point, diminished thermostability [162] and weakened emulsion salt stability. On the other hand, such emulsions show increased storage stability, and therefore, improved shelf-life. According to Sousa et al. [179], zein particles and their complexes can be used as potential stabilizers, inhibiting processes of physicochemical instability, such as chemical degradation, coalescence, Ostwald maturation and lipid oxidation.

In addition, some cultural issues must be solved. Thus, some ingredients or compounds (obtained from the valorization of coproducts) with demonstrated healthy properties may not be widely accepted. Notably, if the sources are not usual in certain diets, e.g., from animal origin (insects and insect protein-rich flours, omega-3 fatty acids from insects, seafood, etc.), which would be especially problematic for vegan consumers [180,181,182].

One important consumer group of fortified foods is female vegan children. Vegan children are at risk of deficiencies of critical nutrients such as protein, long chain fatty acids, cholesterol, iron, zinc, iodine, calcium and vitamins A, B12, and D. Thus, fortified foods may be of particular benefit for them [183].

## 6. Conclusions

The fortification of foods intended for consumption by women is presented as a strategy to reduce macro- and micro-nutrient deficiencies through food innovation, sustainability and gender equity. Numerous foods and their coproducts are excellent sources of nutrients and bioactive compounds such as isoflavones, dietary fiber, folic acid, polyphenols and prenylated flavonoids, which are of great importance in terms of nutrient deficiencies among women.

The female consumer sector, which is particularly preoccupied with the environment and health and safety, would likely embrace the use of sustainable resources from locally produced food processing coproducts to create food fortification compounds to contribute to health and well-being, taking into account the different nutritional requirements associated with various states and/or ages of women (infancy, adolescence, pregnancy, lactation, menopause period and old age). The food industry has an opportunity to align the sustainability of the food production system (SDG 12) with gender equity (SDG 5). These aspects must be associated with Food Product Development and Innovation (FPDI) strategies based on the identification of target groups of women in terms of the nutrients to be added, the selection of foods to be fortified, the development of prototypes and industrial scale up, without forgetting communication and education programs.

In this sense, future research should focus on the appropriate selection of food/coproducts as sources of specific essential nutrients, assessing the feasibility of different food matrixes, selecting fortification technologies and studying the stability, bioavailability and bioaccessibility of essential nutrients in the developed product. Finally, as much as possible, the whole process should be sustainable, ecoefficient and economic.

## Figures and Tables

**Table 1:** Nutritional profile of some cereals, legumes, vegetables, fruits and nuts [45,46,47,48,49].

	Proteins (%)	Fats (%)	Carbohydrates (%)	Dietary Fiber (%)	Vitamins	Minerals
Cereals						
Wheat	14	2.1	60–75 (starch)	11.5–15.5 (Arabinoxylan 5.5–7.4%; β-glucan 0.51–0.96%; Cellulose 1.67–3.05%; Klason lignin 0.74–2.03%)	B_1_, B_2_, B_3_, B_6_, B_9_, E	Ca, P, Mg, Fe, Zn, Cu, Na, K
Rice Bran	10–16	15–22	34–52 (starch)	7–11 Soluble 1.9%	B_1_, B_2_, B_3_, B_5_, B_6_, B_9_, K, tocopherols, and tocotrienols	Cu, Fe, Mg, Se, Zn
Maize	9.5	0.4–17	72 (starch)	9.5 (β-glucan 1.5%)	B_1_, B_2_, B_3_, B_5,_ B_6_, B_9_, E, K	Ca, Cu, Fe, Mg, Mn, P, Se, Na, Zn
Barley	10–20	2–3	54–75 (starch)	11–34 (Soluble 3–20%; β-glucan 2–10%; Arabinoxylan 3.8–6.05%)	B_1_, B_2_, B_3_, tocopherols, and tocotrienols	Ca, P, Mg, Fe, Zn, Mn, Se
Sorghum	6.1–7.3	1.5–2.2	62–67 (starch)	5.4–7.8	Complex B mainly B_6_	K, Mg, Ca, Fe, Zn
Legumes						
Pea	23–26	1.5–2	45–52	11–20 (insoluble 9–16%, soluble 1–8%)	C, A B_1_ and B_3_	Cu, Mn, Fe, Zn
Lentil	26–29	2–2.8	40–46	17–31 (Insoluble 11–17%; Soluble 2–7)	B_1_, B_3,_ B_5_, B_6_ and B_9_	Cu, Mn, Fe, P, and Zn
Common beans	19–24	1.6	45–47	16–25 (Insoluble 10–20%; Soluble 5–10%)	B_9_, B_1_, and K	Cu, Mn, Fe, P, Mg and Zn
Chickpea	23.6	6.4	62 (10.7% sugars)	12–26 (Insoluble 9–19%; Soluble 3–8%)	B_1_, B_6_ B_9_ and choline	Mn, Cu, Fe, Zn, P, Mg, Se and K
Broad beans	27	1.8	35 (5.7% sugars)	20–25	B_9_, K and C	Mn, Mg, P, Cu and Fe
Vegetables						
Potatoes	2.5	0.2	18 (17.1 starch and 0.9 sugars)	2.0	C and B_6_	K, Mg, P, Ca, Fe and Zn
Onions	1.4	0.2	3.5 (0.6 starch and 2.9 sugars)	1.0	A, B_6_, C, E and folic acid	Na, K and Fe
Tomatoes	1	0.1	3.5 (0.1 starch and 3.4 sugars)	1.4	B_1_, B_2_, B_5_, A and C	K, P and Mg
Lettuce	1.5	0.3	1.4 (sugars)	1.5	C and folic acid	P, K, Fe and Ca
Peppers	1	0.4	6.4 (sugars)	1.9	C and A	Fe, Ca, K, I and Zn
Fruits						
Bananas	1.2	0.3	20 (3.1 starch and 16.9 sugars)	3.4	A, C, E and folic acid	K and Mg
Melons	0.6	Tr	6.0 (sugars)	1.0	C and A	K, Mg, Ca and Fe
Apples	0.3	Tr	12.0 (0.6 starch and 11.4 sugars)	2.9	B_1_, B_2_, B_6_, and C	P, K and Ca
Oranges	0.8	Tr	8.6 (sugars)	2.0	C, A and folic acid	K
Grapes	0.6	Tr	15.5 (sugars)	0.4	B_6_, folic acid and K	K, Mn, Mg and Ca
Nuts						
Peanuts	21.6	42.8	0.6	9.5	Niacin, folic acid and pantothenic acid, E and B_1_	Mg, Mn, Cu
Almonds	19.5	43.4	2.1 (sugars)	12.5	E, niacin and folic acid	P, Mg, K, Ca
Walnuts	14.5	64.5	2.1	7.0	B_6_, B_1_, folic acid, B_2_ and B_5_	Cu, P, Mg, Fe and Zn
Cashew	18.9	43.7	4.0	3.3	B_6_ and K	Mg, Mn, P and Zn
Pistachio	19.8	45.1	3.2	7.5	B_6_, B_1_, E, folic acid, B_2_ and B_5_	Cu, P, K, Zn, Ca,

**Table 2:** Nutritional profile of some meats, milk, fish, seafoods and eggs [45,82,83,84].

	Proteins (%)	Fats (%)	Carbohydrates (%)	Vitamins	Minerals	Energy (Kcal/100 g)
Meat						
Pork (lean)	24	3.4 (MUFA 46%, SFA 37%, PUFA 17%; cholesterol 61 mg/100 g)	0.6	B_1_, B*_2_*, B_3_, B_5_, B_6_ and B_12_	Fe, Zn, P, K, Mg, Se	124
Pork (bacon)	9	57 (MUFA 47%, SFA 40%, PUFA 13%; colesterol 100 mg/100 g)	0.2	B_1_, B*_2_*, B_3_, B_5_, B_6_ and B_12_	Na, P, Zn, K, Fe	546
Beef (loin)	21	4 (MUFA 47%, SFA 46%, PUFA 7%; colesterol 80 mg/100 g)	0.8	B_1_, B*_2_*, B_3_, B_5_, B_6_ and B_12_	Fe, Zn, K, P, Mg, Se	112
Beef (skirt)	19	18 (MUFA 51%, SFA 45%, PUFA 4%)	Tr	B_1_, B*_2_*, B_3_, B_5_, B_6_ and B_12_	Na, P, Zn, K, Fe	230
Chicken (Breast)	22	1–2 (MUFA 44%, SFA 31%, PUFA 24%; colesterol 50 mg/100 g)	Tr	B_2_, B_3_, B_6_, B_5_, and B_12_	K, P, Mg Zn and Fe	105
Chicken (skin)	13	35 (MUFA 43%, SFA 35%, PUFA 22%)	Tr	B_2_, B_3_, B_6_, B_5_, and B_12_	K, P, Mg Zn and Fe	362
Milk						
Whole milk	3.3	3.8 (MUFA 26%, SFA 54%, PUFA 20%; cholesterol: 14 mg/100 g)	5	A, D, E and C	Ca, P, K	65
Skimmed milk	3.4	0.1	5	C	Ca, P, K	35
Cream	1.5	48 (MUFA 26%, SFA 54%, PUFA 20%; cholesterol: 140 mg/100 g)	2	A, D, E and C	Ca, P, K	448
Whole milk (goat)	3.4	3.9 (MUFA 27%, SFA 66%, PUFA 7%; cholesterol: 12 mg/100 g)	4.5	A, D, E and C	Ca, P, K	67
Fish and Seafoods						
Salmon (fatty fish)	18.4	12 (MUFA 51%, SFA 20%, PUFA 29%; colesterol 28 mg/100 g)	Tr	A, D, B_3_, B_9_	K, P, Se, Mg, Ca	182
Cod (lean fish)	18	1 (MUFA 25%, SFA 25%, PUFA 50%; colesterol 61 mg/100 g)	Tr		K, P, I, Mg, Se, Ca	83
Swordfish (semi-fatty fish)	18	4.2 (MUFA 48%, SFA 25%, PUFA 27%; colesterol 40 mg/100 g)	Tr	A, D, B_1_, B_3_, B_9_		111
Mussel	10.8	1.9 (MUFA 27%, SFA 32%, PUFA 41%; colesterol 58 mg/100 g)	Tr	B_3_, B_6,_ B_9_	Ca, K, Na, P, Fe, Zn, I	61
Red shrimp	18	1.8 (MUFA 27%, SFA 24%, PUFA 49%; colesterol 185 mg/100 g)	Tr	E, B_3_, B_9,_ B_12_	C, K, Na, P, I, Mg, Fe	90
Eggs						
Whole egg	12.5	11.1 (MUFA 45%, SFA 35%, PUFA 20%; colesterol 385 mg/100 g)	Tr	A, D, E, B_1_, B_2_, B_3_, B_6_, B_12_,	P, Na, K, Ca, Zn	150
Egg white	10.5	0.1	0.3	B_2_, B_3_, B_9_	K, Na, P	45
Egg yolk	16.5	31.5 (MUFA 48%, SFA 36%, PUFA 16%; colesterol 1100 mg/100 g)	2.3	A, D, E, B_1_, B_2_, B_6_, B_12_,	P, Ca, K, Na, Mg	356

## Data Availability

Not applicable.
